# Non-Epithelial Ovarian Cancers: How Much Do We Really Know?

**DOI:** 10.3390/ijerph19031106

**Published:** 2022-01-19

**Authors:** Alison Cheung, Sidrah Shah, Jack Parker, Pavandeep Soor, Anu Limbu, Matin Sheriff, Stergios Boussios

**Affiliations:** 1 Department of Medical Oncology, Medway NHS Foundation Trust, Windmill Road, Kent ME7 5NY, UK; alison.cheung2@nhs.net (A.C.); j.parker14@nhs.net (J.P.); pavandeep.soor@nhs.net (P.S.); a.limbu@nhs.net (A.L.); 2 Department of Palliative Care, Guy’s and St Thomas’ Hospital, Great Maze Pond, London SE1 9RT, UK; sidrah.shah@nhs.net; 3 Department of Urology, Medway NHS Foundation Trust, Windmill Road, Kent ME7 5NY, UK; matin.sheriff@nhs.net; 4 Faculty of Life Sciences & Medicine, School of Cancer & Pharmaceutical Sciences, King’s College London, London SE1 9RT, UK; 5 AELIA Organization, 9th Km Thessaloniki-Thermi, 57001 Thessaloniki, Greece

**Keywords:** germ cell tumours, sex cord-stromal tumours, small cell carcinomas of the ovary, clinical trials

## Abstract

Non-epithelial ovarian cancers (NEOC) are a group of uncommon malignancies that mainly includes germ cell tumours (GCT), sex cord-stromal tumours (SCST), and some extremely rare tumours, such as small cell carcinomas and sarcomas. Each of these classifications encompasses multiple histologic subtypes. The aetiology and molecular origins of each sub-group of NEOC require further investigation, and our understanding on the genetic changes should be optimised. In this article, we provide an update on the clinical presentation, pathology, genetics, treatment and survival of the main histological subtypes of the GCT and the SCST, as well as of ovarian small cell carcinomas. We also discuss miRNA expression profiles of NEOC and report the currently active clinical trials that include NEOC.

## 1. Introduction

Epithelial ovarian cancer (EOC) is the most lethal gynaecologic malignancy, as it is commonly diagnosed at an advanced stage. Non-epithelial ovarian cancers (NEOC) are rare, accounting for approximately 10% of all ovarian cancers and include mainly germ cell tumours (GCT), sex cord-stromal tumours (SCST), and some extremely rare tumours [1,2,3]. The greatest risk factors of ovarian cancer are a family history and associated genetic syndromes. Several modifiable risk factors, such as obesity, smoking and sedentary lifestyle, are associated with an increased risk of ovarian cancer, but have not been established as predisposition factors. Endometriosis is directly related to certain EOC subtypes, specifically clear cell and endometrioid ovarian carcinoma, rather than to NEOC [4].

GCT originate from the primordial germ cell and are divided into dysgerminomas and nondysgerminomas, including primarily yolk sac tumours (YST) and immature teratomas. GCT most often occur among younger women of childbearing age. However, cases of postmenopausal GCT, primarily YST, have been reported in the literature. SCST arise from the sex cord and ovarian stroma and comprise a heterogeneous group of tumours, of which granulosa cell tumour (GrCT) is the most frequent type. SCST are mainly diagnosed in older age groups with a median age of around 50 years. GrCT are divided into adult and juvenile types, of which the adult type represents approximately 95%. The *Forkhead box L2* (*FOXL2*) gene mutation has been found to be an extremely sensitive and quite specific marker for the adult GrCT.

The initial symptoms and signs of NEOC are usually a subacute pelvic pain, feeling of pelvic pressure due of a pelvic mass and menstrual irregularities [2]. Symptoms become more noticeable as the cancer progresses. Diagnostic work-up should include pelvic ultrasound, an abdomino-pelvic computed tomography (CT) scan, chest X-ray and a positron emission tomography (PET) scan in selected cases. From the biochemical perspective, serum beta-hCG (β-hCG), α-foetoprotein (AFP) and lactate dehydrogenase (LDH) levels, along with liver and renal functions, should be carried out. Full blood count tests are also recommended. Inhibin B is secreted by GrCT and could be a useful marker for the disease. Serum anti-Müllerian hormone (AMH) may be a marker of ovarian reserve and GrCT in postmenopausal or post-oophorectomy women. While these markers are nonspecific, they can provide prognostic information, and as such quantitative hCG, AFP, LDH and cancer antigen 125 (CA-125) should be measured preoperatively [2]. Fertility-sparing surgery (FSS) and platinum-based chemotherapy remain the standard of care, providing a high chance of cure at all stages. Given the lack of high-quality studies in this field, current practice guidelines recommend chemotherapy regimens adopted in testicular GCT. However, platinum-resistant/refractory GCT retain a worse prognosis in comparison with their male counterpart.

This review summarises the published literature on the clinical, histological and therapeutic aspects of the main subtypes of NEOC, along with miRNA expression profiles and clinical trials that currently recruit patients.

## 2. Ovarian GCT

Ovarian GCT originate from the primitive germ cell of the embryonic gonad and account for around 1–2% of all ovarian cancer [5,6,7]. GCT are diagnosed principally in the first three decades of life. Siegel et al. found 3% of cases occurring from birth to age 14 and 11% of cases in those aged 15 to 19 [8]. The World Health Organisation (WHO) has classified ovarian GCT histologically into three categories—primitive GCT, teratomas and monodermal and somatic-type tumours arising from dermoid cysts. These are summarised in Table 1 [2,5,9,10]. GCTs differ to EOC with their earlier age of incidence, faster rate of growth, unilateral localisation (95% of cases) and good prognosis [11,12,13]. Teratomas are a common GCT with the majority characterised as having mature differentiated tissues [5]. Mature teratomas are benign in all females regardless of age, whereas immature teratomas are malignant and grading is significant prognostically [2,5]. Grading of immature teratomas is from 1 to 3 and measured by the amount of immature neuroepithelial tissue per low power field [5]. Primitive GCT and immature teratomas have high chemosensitivity and are treated with FSS; therefore, histological diagnosis is crucial to determine the appropriate treatment option [2]. Up to 2018, 193 patients were diagnosed with gestational NEOC. Among 145 documented cases of GCT, histopathology was compatible with endodermal sinus tumour in 52 patients, and dysgerminoma in 45 patients [14]. The optimal timing of surgery is at midgestation, whereas chemotherapy can be administered during the second and third trimesters.

### 2.1. Dysgerminomas

Dysgerminomas are the most common GCT and can present at any age, but most of them occur in adolescence and early adulthood [15], with 85% of patients aged less than 30 years at time of diagnosis [16]. Patients will often present with abdominal pain, distension, and menstrual disorders [6]. There is nonspecific elevation of serum alkaline phosphatase (ALP) and lactic dehydrogenase, and 5% of dysgerminomas secrete β-hCG because they contain multinucleated syncytiotrophoblastic giant cells [6]. Dysgerminomas are solid and well encapsulated large masses, which on sectioning will reveal a soft, fleshy, and lobulated tan or grey surface [6]. Rarely would they cause haemorrhage or necrosis [10]. Histologically, uniform, large tumour cells are separated by fibrovascular septae which is almost always infiltrated with chronic inflammatory cells, especially lymphocytes [10,17]. This appearance is reflected on various modes of imaging. On ultrasound they will appear as smooth, well-defined lobules with heterogenous echogenicity and will be vascularised on colour and power Doppler ultrasound [18]. Furthermore, on CT and magnetic resonance imaging (MRI), a solid tumour will be seen with the fibrovascular septa along with areas of necrosis or haemorrhage if present [6,17]. Immunohistochemistry staining may also be positive for markers, such as placental alkaline phosphatase (PLAP), cluster of differentiation 117 (CD117) and D2-40, with *c-KIT* mutation being present in 33–50% of patients [10,19]. Dysgerminomas have a favourable prognosis, with a five-year survival of approximately 90%; this decreases to 63% if the disease has extended beyond the ovaries [10,20]. Recurrence rates may range between 18–52%, with over 75% of these occurring in the first year after diagnosis [7,10].

### 2.2. Yolk Sac Tumours

**Figure 1:**
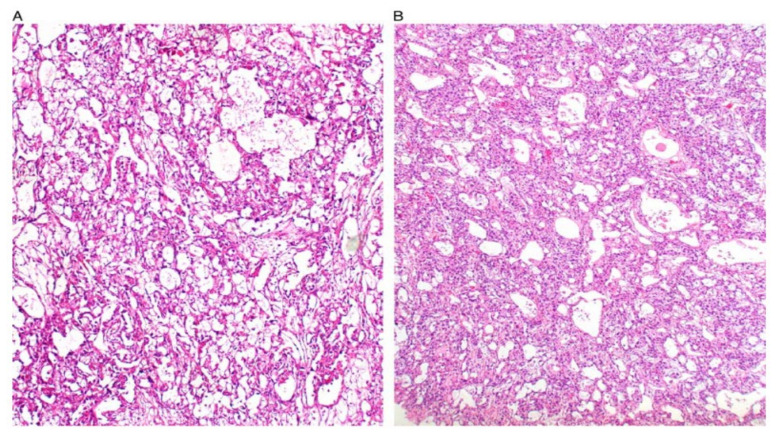
Reticular and microcystic (reticulocystic) patterns (**A**). Characteristic meshwork of spaces merging with cysts of varying sizes and shapes (**B**).

**Figure 2:**
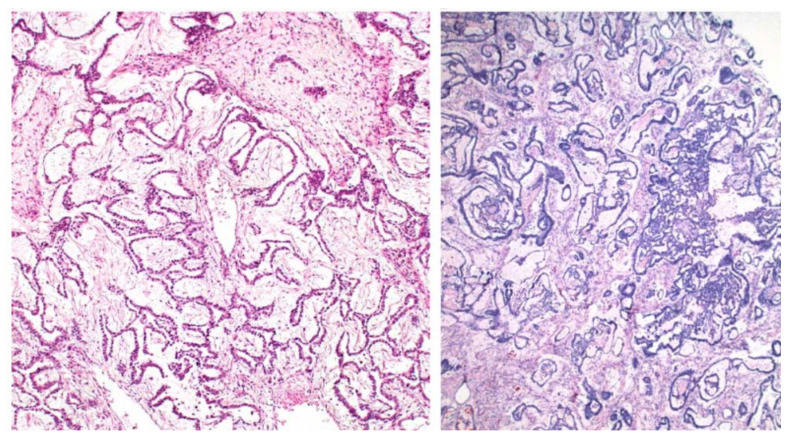
Festoon pattern with arching drape-like configurations.

**Figure 3:**
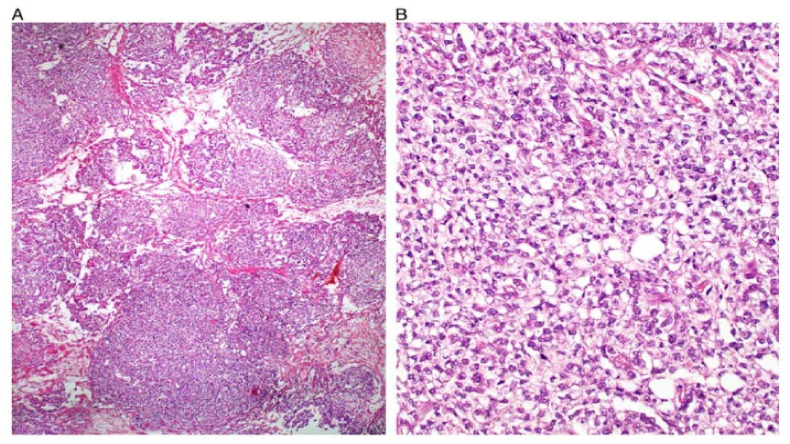
Solid pattern. Large focally coalescent aggregates are seen (**A**). Note typical abundant pale to clear cytoplasm and nuclei that are smaller than those of dysgerminoma (**B**).

**Figure 4:**
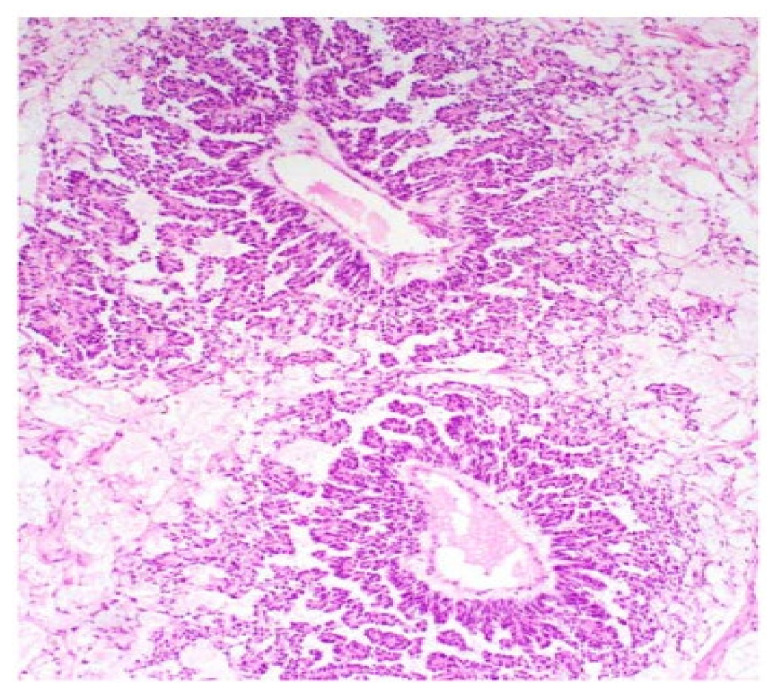
Papillary pattern. Occasionally the characteristic papillae radiated, like the spokes of a wheel, from a large dilated vessel.

**Figure 5:**
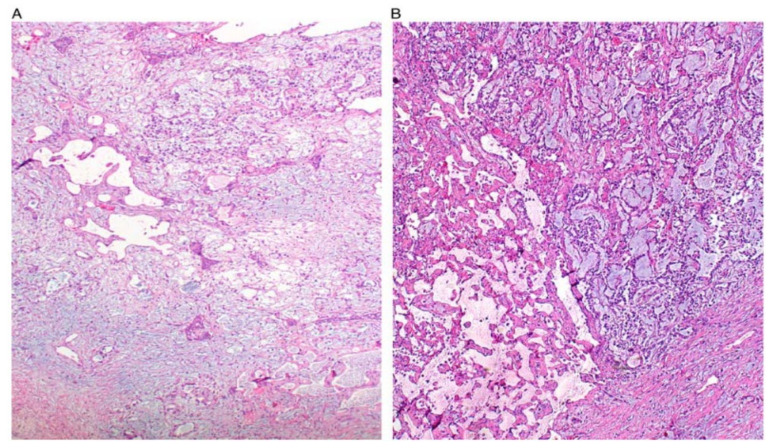
Myxoid appearance. Myxoid matrix occupying much of the stroma (**A**) and similar material within cyst lumens (**B**).

YST are the third most common GCT and mainly occur in women in their second and third decades of life [10,21]. YST is extremely rare in menopausal women but case reports of this are documented in the literature, with ages ranging between 50 and 86 years, and poorer outcomes have been identified for this demographic [22]. Patients will present with abdominal pain, a palpable abdominal mass, or an acute abdomen secondary to ovarian torsion [10]. Unlike dysgerminoma, serum AFP and CA-125 will be elevated in most patients [23]. Grossly, YST are unilateral (mostly), large, and well-encapsulated tumours, which have a mixture of solid and cystic components and areas of haemorrhage or necrosis [6]. The cysts vary in size and will be scattered throughout to give a wet honeycomb appearance [6]. The structure of YST resemble the primitive yolk sac and they have a variety of histological appearances, which are summarised in Table 2 [6,10,24]. Some figures are also provided (Figure 1, Figure 2, Figure 3, Figure 4 and Figure 5). On CT and MRI imaging, YST will appear as a solid cystic mass with areas of haemorrhage and capsular tears may also be present [6,25]. Immunohistochemical staining for AFP is characteristic of YST [22]. YST is a highly malignant tumour which invades local structures and metastasises rapidly to intra-abdominal structures and retroperitoneal lymph nodes [10,26,27]. However, the combination of surgery with chemotherapy has now led to an overall cure rate of 80% [7,10,28].

### 2.3. Treatment of GCT

Most GCT will be diagnosed at early stages and FSS is the main treatment option, especially as the patients affected are children or young women [13,29]. This includes unilateral salpingo-oophorectomy (USO) with the unaffected ovary and uterus left in place—biopsy of the second ovary is only performed if there is obvious abnormality to reduce the risk of adhesions or ovarian failure [6,10]. FSS requires further prospective evaluation, but multiple case studies have demonstrated successful outcomes with USO when disease is limited to one ovary [30,31]. Furthermore, peritoneal surfaces, the omentum, and lymph nodes (retroperitoneal and ipsilateral pelvic) are thoroughly examined and resected or biopsied if any abnormalities are present [6,32]. Surgical staging also includes peritoneal washings and ascites sampling if present [10]. If advanced disease is present, debulking surgery is performed instead to try and remove as much cancer as possible and second-look surgery may be an option if the cancer is not resected completely [10].

Depending on the histology, staging and molecular features of the GCT, surgery will be followed by active surveillance or adjuvant chemotherapy, which has revolutionised GCT treatment in the last 40 years [5,6]. Currently, dysgerminomas confined to the ovary and grade I immature teratoma are managed with surveillance post-operatively [5]. Otherwise, platinum-based chemotherapy is used, and the standard regime consists of bleomycin, etoposide and cisplatin (BEP) for four to six cycles [6,33]. Survival rates with BEP range between 82% to 100% in early-stage disease and 75% in advanced-stage disease [34]. There is concern regarding long-term toxicity of chemotherapy, but Kang et al. showed that this regime did not impair ovarian function and fertility [33]. While women stopped menstruating during chemotherapy, over 85% have been shown to regain menstrual function once chemotherapy was completed [35,36,37]. These findings are reassuring that fertility can be preserved in women completing GCT treatment with successful pregnancies shown to match those of the general population [5]. A subset of GCT can acquire *KRAS*-activating mutations and other genetic alterations, such as *BRCA1/2*, *KIT*, and *MAPK*; nevertheless, the efficacy of targeted therapy and genomic features contributing to chemoresistance still remain to be elucidated. Finally, programmed death-ligand 1 (PD-L1) overexpression in testicular GCT has been recently described. However, the preliminary results were not suggestive of therapeutic efficacy by immune check point inhibitors in molecularly unselected patients [38].

Recurrences usually occur within two years of initial diagnosis and typically relapse in the peritoneal cavity and retroperitoneal lymph nodes. The response rate to salvage chemotherapy in patients with GCT is approximately 50%, and recommended regimens include vinblastine, ifosfamide, and cisplatin; etoposide, ifosfamide, and cisplatin; and paclitaxel, ifosfamide, and cisplatin. Secondary cytoreductive surgery could be performed in selected patients with recurrent disease [39].

## 3. Ovarian SCST

Ovarian SCST include both benign and malignant cancers which originate from either the sex cord or stromal cells, or both. As such, different examples of these SCST include pure sex cord tumours (including juvenile and adult GrCT), pure stromal tumours (including fibromas) and mixed SCST, including Sertoli-Leydig cell tumours (SLCT).

SCST occur in different age groups, with GrCT occurring mainly in women who are peri- or post-menopausal, and SLCT largely occurring in young females. The yearly-adjusted incidence rate of SCST is 2.1 per 1 million women [2,40].

Generally, SCST show no association with breast cancer, except for GrCT, which may occur at a higher frequency in patients with a *BRCA* mutation or who have a history of breast cancer [41]. Major developments have identified the *FOXL2* and *DICER1* mutations in adult GrCT and SLCT respectively. These can help in the diagnostic process, and may have a role in prognostication.

Treatment of SCST can involve surgery, chemotherapy, radiotherapy and targeted therapy. Cytoreductive debulking surgery is the standard management, which may be followed by adjuvant platinum-based chemotherapy. In advanced disease, if there is tumour recurrence, cytoreductive surgery can be used. Complete resection of the tumour during the initial surgery is important, as it has been shown in that for those patients who had residual tumours and underwent cytoreductive surgery, they all developed tumour recurrence. The differential diagnosis for SCST can include such diverse entities as carcinoma, sarcoma, GCT, melanoma, peritoneal carcinomatosis and malignant peritoneal mesothelioma [40,42].

### 3.1. Ovarian GrCT

GrCT account for up to 5% of overall ovarian cancers. There is an incidence of 0.61 cases per 100,000 women per year, accounting for around 2–5% of all ovarian cancers [43]. Adult GrCT are the most common form, comprising around 95% of GrCT. The majority of these present at an early stage, in an indolent fashion.

The most common presenting complaints with SCST are the presence of an adnexal mass, abdominal pain and distention. Some of these tumours are termed “functioning” and can release hormones, including androgens and oestrogens. As such, patients may have evidence of oestrogen excess, including abnormal vaginal bleeding, precocious puberty, or androgen excess, including hirsutism [44,45].

The presence of some tumour markers can be of diagnostic benefit. Oestradiol levels can be measured and also manifest with clinical signs and symptoms, as mentioned earlier, but in around 30% of GrCT, oestradiol is not secreted so it cannot be used as a marker of disease activity [46].

Inhibin A and B, oestradiol and MIH (Müllerian inhibiting substance) are hormones secreted by GrCT. Inhibins are secreted by ovarian granulosa cells and are part of a negative feedback loop which inhibits the pituitary secretion of the follicle stimulating hormone (FSH). In postmenopausal women, the levels of inhibin should be undetectable, but can be elevated in instances of GrCT. Inhibin B is more associated with disease progression than inhibin A [47]. MIH levels can also be elevated, and the combination of inhibins and MIH is useful for monitoring disease status.

Adult GrCT have been found to have an association with a somatic *c.402C* > *G* missense point mutation in the *FOXL2* gene. This is pathognomonic for adult GrCT and may have a causative role [48]. In addition, genetic studies can be used to differentiate between early stage and advanced stage adult GrCT.

Due to oestrogen production by GrCT, there can be an associated finding of endometrial hyperplasia, and endometrial carcinoma can be seen in up to 10% of patients with GrCT. These are often at an early stage, and thus carry a good prognosis [49].

Surgical management is used to treat GrCT. Patients who present in their reproductive years are managed with a USO. For patients who are postmenopausal or have no desire of childbearing, they are treated with a total abdominal hysterectomy and bilateral salpingo-oophorectomy (BSO) [45]. For patients with stage IA GrCT, surgery alone provides an excellent prognosis, with no adjuvant therapies used. Around 97% of GrCT are unilateral [50]. For stage IB, i.e., involving both ovaries, a total abdominal hysterectomy with BSO is used. FSS can be attempted in selected cases. For stage IC, surgery alongside adjuvant platinum-based chemotherapy is used. For advanced or metastatic disease, cytoreductive surgery is the most effective treatment. Additional chemotherapy courses can be used in these patients, as the prognosis is poor, with a high recurrence rate [2].

Hormone therapy has been shown to have a role in GrCT which express steroid hormone receptors. No corroboration between hormonal therapy and hormone receptor expression has been established. Aromatase inhibitors have been found to be the most responsive hormone therapy, though data is limited [51].

GrCT have a good prognosis as they are usually diagnosed at an early stage. The median five-year survival has been reported to be 95% for stage I and 59% for stage III/IV disease [52]. There has been a dose-dependent gonadotoxic response found to cisplatin-based chemotherapy, which has also been found in males. As such, the possible treatment outcomes should be weighed against the possible long-term adverse effects. In such patients, oocyte preservation can be undertaken, though the rate of infertility in patients who have attempted conception after this chemotherapy has not been shown to be any higher than the baseline infertility rate for the general population [2,53].

There are numerous significant prognostic factors related to GrCT, including the stage of the tumour, size of the tumour, whether or not it has ruptured, the mitotic index, and the presence of residual disease following surgical resection and grading. The most accurate prognostic factor in GrCT is the staging of the tumour at the time of diagnosis [52]. An early stage is also associated with reduced recurrence rates [45].

Juvenile GrCT account for a very small proportion of GrCT (around 5%). However, in prepubertal patients and in women less than 30 years of age, juvenile GrCT accounts for around 90% of cases [54]. As in adult GrCT, juvenile GrCT commonly presents with abdominal pain and an adnexal mass. However, most are unilateral and present at an early stage. The tumour grows as a solid mass. These tumours are often functional, secreting oestrogen, which causes precocious puberty in around 80% [40]. There may also be secretion of testosterone, causing virilisation in some patients.

Juvenile GrCT do not express a *FOXL2* mutation, which can help in the diagnostic process. Histologically, juvenile GrCT have a distinct appearance with Call-Exner bodies, and pale, round nuclei with a low mitotic rate. This is in contrast to adult GrCT, which do not have such typical histologic findings [55]. Progesterone levels and inhibin levels can be elevated in juvenile GrCT.

Since most juvenile GrCT present at an early stage, management is largely surgical and curative. Since there may be endometrial hyperplasia (if there is an oestradiol excess), an endometrial biopsy should be taken, particularly since a total abdominal hysterectomy is usually not necessary.

Juvenile GrCT are usually diagnosed at an early stage (IA) and since the tumour is indolent and usually has a higher mitotic rate, the prognosis is very good. Recurrence beyond three years post-surgery is rare [56]. The median five-year disease-free survival rate post-surgery has been estimated at 92% [54]. As in adult GrCT, if the disease is at a later stage, the prognosis is worse. The relapse rate is higher and the natural history of the disease is more aggressive.

### 3.2. Sertoli-Leydig Cell Tumours

SLCT are rare; they account for less than 0.5% of ovarian tumours. The majority occur in women aged between 20 and 40. Their specific incidence is not easily determined. They often present with a unilateral mass. The tumours are usually hormone-secreting; the majority secrete testosterone but some may produce oestradiol. Up to 85% of patients experience virilisation. Patients may have pure Sertoli cell tumours which can secrete renin, which can cause hypertension [40].

SLCT are formed of varying proportions of Sertoli cells and Leydig cells, and may have other heterogeneous elements [56]. These cells are normally found around the seminiferous tubules of the testicles, and are involved in androgen production. SLCT are classified into well-differentiated, moderately differentiated and poorly differentiated tumours. Both moderately- and poorly-differentiated SLCT express at least mutation in the *DICER1* gene, whilst well-differentiated SLCT can be *DICER1*-independent. The association between the *DICER1* mutation and SLCT has been shown to be around 88% [57]. The *FOXL2* missense mutation, which is associated with GrCT can also be found in SLCT, but the sensitivity is only around 50%. As such, *FOXL2* is generally used with serum inhibins to distinguish SCST from non-SCST, rather than to compare different SLCT [58].

For young patients who have stage IA SLCT and are of reproductive age, FSS is offered. Those with poorly differentiated malignancy are offered adjuvant chemotherapy. Patients who have no desire of childbearing are offered a total abdominal hysterectomy and BSO. For SLCT staging greater than IA, surgery and adjuvant chemotherapy are offered irrespective of tumour differentiation. The chemotherapy regimens are platinum-based [2].

SLCT carry a good prognosis since most patients at time of diagnosis have a disease stage of I; nevertheless, advanced stages carry an extremely poor prognosis [59].

## 4. Small Cell Ovarian Carcinomas

Small cell carcinomas of the ovary hypercalcaemic type (SCCOHT) typically occur in adolescents and young women with a peak incidence in the third decade of life. Serum calcium levels may serve as a marker for treatment response and recurrences. The pathophysiology of hypercalcemia is unclear, it has been postulated that the parathyroid hormone or parathyroid hormone-related protein (PTHrP) appear to be crucial [40]. The prognosis of SCCOHT is very poor and the risk of extra-ovarian spread high [2].

A germ cell origin for SCCOHT has been suggested. However, more recently, SCCOHT have been sequenced and confirmed to be a malignant rhabdoid tumour by virtue of consistent deleterious mutations in *SMARCA4*, a chromatin-remodelling gene encoding protein BRG1. These tumours resemble high grade neuroendocrine histological features; nevertheless, they are now recognised to be a distinct clinical and pathological entity. They are predominantly characterised by variable numbers of larger cells with a luteinised or rhabdoid appearance [40].

There is currently no consensus as far as the treatment of the SCCOHT is concerned. A combination of treatment modalities, consisting of debulking surgery, followed by chemotherapy and possibly radiotherapy is recommended. FSS is reasonable, as the disease is mostly unilateral [40]. A combination of a cisplatin and etoposide-based therapy is generally considered most appropriate. SCCOHT are particularly chemosensitive at the outset but the relapse is usually rapid.

Small cell carcinomas of the ovary, pulmonary type (SCCOPT) affect older, peri- or postmenopausal patients. In contrast with SCCOHT, they are not correlated with hypercalcemia. SCCOPT are predominantly unilateral and have dismal prognosis even when diagnosed early. Histopathologically, they have solid growth of small cells arranged in sheets and closely packed nests. The tumour cells are pleomorphic, round to spindle-shaped, with scanty cytoplasm and hyperchromatic and elongated nuclei. Pre-existing endometrioid carcinomas and Brenner tumours are predisposition factors for the development of the SCCOPT. Neuroendocrine markers (chromogranin, synaptophysin and NSE) are diffusely positive by immunohistochemistry [40].

Conventional surgical treatment includes radical surgery (hysterectomy and BSO), followed by adjuvant platinum-etoposide chemotherapy. The evidence for the potential role of the immunotherapy is extrapolated from the small-cell lung cancers. It seems that there is a promising activity of the anti-PD1 antibodies in this context [2].

## 5. mRNA Profiles of NEOC

MicroRNAs (miRNA) are endogenous RNAs that are transcribed from DNA sequences, made up of approximately 18–25 nucleotide non-coding RNAs. There are estimated to be more than 2600 miRNAs encoded in the human genome [60]. Through degradation of target miRNA and inhibition of protein translation, miRNA regulate gene expression [61]. Research over the last two decades has facilitated a greater understanding of the significance of miRNA in the pathogenesis of cancer. Through various mechanisms involving the loci of miRNA, alterations in the expression of miRNA cause dysregulation of oncogenes and tumour-suppressor genes, and in turn drive malignant cells [62]. Identification of miRNA can provide valuable information in diagnosis and prognosis and offers predictive value in several malignant diseases, such as colorectal and prostate cancers, melanomas and carcinomas of unknown primary [63,64,65,66].

As NEOC is less common than EOC, very little is understood about its molecular pathogenesis. At present, biomarkers used in diagnosis and monitoring for NEOC and, in particular GCT are neither sensitive nor specific [61]. Further understanding of miRNA profiling in NEOC could allow for development of the diagnosis, prognosis, monitoring and targeted treatment of the malignant disease.

There has been an increasing body of literature assessing the transcription factors expressed in GrCT and SCST. FOXL2 is a marker of ovarian differentiation and is involved in granulosa cell development [67]. RNA sequencing revealed recurrent somatic mutations (*c.402C > G*) of *FOXL2* in an analysis of four adult GrCT [48]. When compared with its wild-type, mutant *FOXL2* was defective in promoting granulosa cell death [68]. C134W mutation of *FOXL2* also promotes oncogenic activity in GrCT [69]. DICER1 is an endoribonuclease involved in miRNA processing. It functions in the RNA interference pathway to cleave long double-stranded RNA molecules into short ones, known as small RNAs, including miRNA and small interfering RNA. *DICER1* mutations were found in 60% of Sertoli-Leydig cells, and the oncogenic effects could be due to a disruption of miRNA processing [70]. 

Various controllers of pluripotency are expressed in GCT. Lethal-7 (let-7) is a group of nine miRNA that function as important tumour-suppressor genes [71]. Let-7 is negatively regulated by the RNA-binding protein LIN-28 homolog A (LIN28), which controls the pluripotency of embryonic stem cells. LIN28-positive GCT have been shown to have reduced levels of let-7 miRNA, therefore suggesting that the LIN28/let-7 pathway could have a significant role in the pathogenesis of GCT [72,73]. Spontaneous mutations of *KIT* proto-oncogene have also been shown to cause undifferentiated oogonia proliferation in 60 GCT samples [74]. Studies assessing both male and female GCT have demonstrated expression of other mediators of pluripotency, such as *NANOG* and *POU5F1* [75].

When compared with other malignancies, there have been significantly fewer studies examining the miRNA expression profiles of NEOC. Whilst previous studies have found that there are alterations in the expression of miRNA clusters in GCT, they have often been carried out in combination with testicular GCT [76,77]. One review paper, which compared the miRNA profiling of ovarian and testicular GCT, identified clusters mir-302~367 and mir-371~373 as overexpressed in GCT, from various studies. Gene ontology has demonstrated the significance of these overexpressed miRNA clusters, by revealing that they act to down-regulate mRNA that have oncogenic importance, and that they appear specific for malignant GCT [77]. These clusters have been demonstrated as potential markers for malignant GCT diagnosis and monitoring through a case study of a paediatric YST [61].

Whilst the aforementioned are all examples of miRNA profiling assessing both male and female germ cell tumours, mir-302 and cluster mir-371~373 were also demonstrated as highly expressed in malignant GCT in a study using small RNA sequencing of nine benign and malignant GCT and three SCST [78]. It also found lower expressions of mir-199a-5p in malignant GCT, when compared with benign GCT and SCST. Mir-199a-5p is a known down-regulator of the *autophagy gene beclin 1* (*BECN1*) [79]. *BECN1* was also highly expressed in malignant GCT, leading the authors to postulate that autophagy might have an oncogenic role in GCT [78]. When assessing SCST, mir-202-3p and mir-513c-5p were found to be considerably higher than in malignant and benign GCT. Given that mir-202-3p is expressed in sertoli [80] and granulosa cells [81], the authors reasonably proposed it as a specific marker for SCST [78]. Mir-202-3p is correlated with expression of the transcription factor FOXL2 [82]. Studies have shown recurrent somatic mutations in *FOXL2* in GrCT of the ovary [48]. The precise relationship with the mir-202-3p/FOXL2 pathway and the development of SCST is currently not well understood. Little is known about mir-506~514 in the context of ovarian cancer. Given that the metabolic function modulator FOXO1 transcription factor induces the mir-506~514 cluster [83,84], and that impaired function of FOXO1 has been linked with GrCT development in mice [85], Chang et al. speculated that the mir-506~514 cluster and its relationship with FOXO1 may play a role in GrCT development [78].

From the relevant literature, it is clear that there exists significant and specific expression patterns of miRNA in NEOC. Previously, research has included both testicular and ovarian tumours and this may largely be due to the rarity of NEOC. Studies, such as the one undertaken by Chang et al. [78], which assessed miRNA profile characterisation via sRNA sequencing, and used only NEOC samples, are very beneficial in providing a basis for future studies to further explore the specific mechanisms by which these miRNA clusters are involved in the pathogenesis of NEOC. Table 3 depicts the main miRNAs and their targets in NEOC.

## 6. Clinical Trials and Novel Approaches of NEOC

According to the European Society for Medical Oncology (ESMO) guidelines, the current standard for post-surgical treatment for various forms of NEOC is platinum-based chemotherapy, with BEP being the most widely-used regimen [2]. However, BEP has many non-negligible side effects. Moreover, one-third of patients relapse or fail to respond after the first-line chemotherapy. A fraction of these patients would subsequently develop platinum-resistant disease, which is when they develop serological or radiological disease progression within four weeks of prior platinum-based regimen [86].

Administering standard chemotherapy 2- rather than 3-weekly (“accelerating chemotherapy”) has been proven to improve cure rates in other cancers. Following a phase II trial with promising results, there is an ongoing international phase III trial investigating whether accelerated BEP chemotherapy is more effective than standard BEP chemotherapy in adult and paediatric males and females with intermediate and poor-risk metastatic GCT (NCT02582697). The primary endpoint is progression-free survival, and secondary endpoints are response following treatment completion, adverse events, health-related quality of life, treatment preference, delivered dose-intensity of chemotherapy, overall survival and associations between biomarkers (to be specified) and their correlations with clinical outcomes [87].

The role of active surveillance in early-stage NEOC in the paediatric/adolescent cohort is unclear, due to the paucity of randomised studies [88]. There is an ongoing phase III trial with a large estimated enrolment size that studies the effectiveness of active surveillance, bleomycin, etoposide, carboplatin or cisplatin in treating paediatric and adult patients with GCT in both genders (NCT03067181). Patients will undergo assessment of their disease risk, and be randomised into either BEP chemotherapy or active surveillance. This trial has the primary outcome of overall survival and event-free survival over two years post-enrolment. It also has other outcome measures of percentage of patients with hearing loss and peripheral neuropathy. The results of the study would potentially change the standard management strategy for these patients.

The Gynaecologic Oncology Group (GOG) has completed a phase II trial that assesses the clinical response and safety of paclitaxel as a single agent therapy in patients with measurable malignant SCST who had received only one prior chemotherapy regimen [89]. Measurable disease was defined by the GOG Response Evaluation Criteria in Solid Tumours (RECIST) [90]. Among 31 enrolled women, one achieved complete response and eight partial responses. Another objective of this study was to evaluate the value of inhibin A and B as a predictor for treatment response. The rationale behind that was that there had been previously performed studies, which showed that increased level of serum inhibin B at the initial diagnosis of GCT predicts relapse before the manifestation of clinical symptoms [91]. The pre-treatment inhibin was not elevated in the majority of patients enrolled in the study, which concluded that it is not a reliable tumour marker. As a continuation of this clinical trial, the GOG group is leading a phase II trial on the effectiveness of BEP versus the combination of paclitaxel and carboplatin for newly diagnosed advanced or recurrent chemo-naïve ovarian GCT, SCST, SLCT, gynandroblastomas and steroid cell tumours (NCT01042522). It also has the tertiary objectives to collect tumour tissues for future translational studies and to examine the changes of inhibin A and B levels in these patients pre- and post-treatment.

In patients with refractory testicular GCT, high-dose chemotherapy (HDCT) with autologous stem-cell transplantation (AuSCT) is one of the salvage treatment options. The role of AuSCT in NEOC remains ambiguous, due to the lack of prospective data [2]. A randomised phase II trial of maintenance etoposide versus observation in relapsed GCT patients who had HDCT and peripheral stem-cell transplant within 16 weeks is currently recruiting patients of both genders (NCT04804007). The primary outcome is 12-month progression-free survival, and the secondary outcome is 12-month overall survival, along with the toxicity and tolerability of maintenance etoposide. The experimental arm would have maintenance daily oral etoposide 50 mg/m^2^ for 21 days in a four-week schedule, for a total of three cycles. Patients randomised to the other arm would be observed with regular follow-ups. Outcomes of this study would be pivotal in determining the role of HDCT with AuSCT in refractory ovarian GCT.

Currently, the corroboration between hormone receptor expression in adult GrCT and hormonal therapy has yet to be established, while there is some data showing GrCT response to aromatase inhibitors [2]. There is a basket phase II trial examining the effect of oral progesterone antagonist onapristone ER (extended release) alone or in combination with anastrozole in women with progesterone receptor (PR) positive adult GrCT, low grade serous EOC or endometroid endometrial cancer (NCT03909152). For PR positive GrCT, the arm for onapristone ER as a monotherapy is closed. The results of this trial would provide more data on the use of hormonal therapy in GrCT.

Immune-checkpoint inhibitors have also been explored as a therapeutic option in malignant ovarian GCT. Programmed death-1 (PD-1) and PD-L1 expression are reported in 75–80% of SCST and also in a majority of testicular GCT [92,93]. However, the preliminary studies of PD-L1 inhibitor monotherapy against relapsed GCT in male patients did not show promising results. Adra et al. conducted a single-arm phase II trial using pembrolizumab 200 mg every three weeks, until disease progression in 12 adult patients with refractory GCT who were resistant to cisplatin-based chemotherapy and after at least one salvage regimen [94]. All patients had nonseminoma. Two patients achieved radiologically stable disease for 28 and 19 weeks each, respectively. Both patients had a continued rising AFP level, despite radiographic stability, and had negative PD-L1 staining. No immune-related adverse events were reported. Another phase II study investigated the efficacy and safety of avelumab in the dosage of 10 mg/kg administered biweekly to eight patients with multiple relapsed and/or refractory GCT. Among them, five were completely refractory to cisplatin [95]. No severe adverse events were observed. All patients showed disease progression at the median follow-up of 2.6 months.

A phase 2 trial studying the efficacy of nivolumab (PD-L1 inhibitor) and ipilimumab (CTLA-4 inhibitor) on adult patients with rare tumours is currently recruiting (NCT02834013). It includes SCCOHT and ovarian GCT. Patients are assigned to either one of two arms. The first arm includes all cohorts, except the PD-L1 amplified cohort, which will receive ipilimumab plus nivolumab combination therapy. Arm II incorporates the PD-L1 amplified cohort, which will receive nivolumab only. The primary objective is the overall response rate of each subset of cancer, and the secondary objectives include toxicities, overall survival and progression-free survival of each cohort. This study outcome will be important in the evaluation of using immune-checkpoint inhibitors to treat GCT and SCCOHT.

The APACHE study is an open-label, randomised, parallel arm, phase II trial of durvalumab alone (arm A) or in combination with tremelimumab (arm B) in patients with advanced and relapsed GCT [96]. Twenty-two patients were randomised with 11 patients per arm. In stage I, one patient in arm B experienced a partial response of multiple lung metastases, and another patient in arm B achieved stable disease with a decrease in serum markers. Arm A was closed due to accrual, as eight of the patients demonstrated hyper-progression features. The second stage of the APACHE trial does not only continue to study the efficacy of combination therapy in relapsed/refractory GCT, but also includes both female and male adult patients. This currently recruiting study has the primary outcome of overall response rate within a year (NCT03158064). Given the encouraging results from stage I, the outcome of stage II would be pivotal to the use of combination immunotherapy in relapsed/refractory GCT and its predictive biomarkers.

Currently active clinical trials that include NEOC are shown in Table 4.

## 7. Conclusions and Future Directions

NEOC represents a group of rare ovarian cancers, which mainly affects young women and adolescents. Each cancer has a distinct clinical, histological and micro-RNA expression profile. Molecular profiling of tissue samples from different forms of NEOC helped to identify mutations that are highly prevalent to the cancer subtype. Further research is required to elucidate their potential as therapeutic targets. Examples include *FOXL2* in adult GrCT, *AKT1* and *GNAS* in juvenile GrCT, *DICER1* and PD-L1 in SLCT and *SMARCA4* in SCCOHT.

The main treatment strategy is surgical resection, followed by a platinum-based chemotherapy regimen. There are ongoing clinical trials studying different combinations of chemotherapy agents and also exploring novel biological agents against NEOC. Due to the rarity of these tumours, randomised trials assessing the effectiveness of different treatment options are often difficult. Patients diagnosed with NEOC should be referred to tertiary centres and actively included in clinical trials at the earliest opportunity. International collaboration is crucial in advancing our knowledge and achieving a standardised and evidence-based management strategy for NEOC.

## Figures and Tables

**Table 1:** World Health Organisation classification of GCT [2,5,9,10].

**Primitive GCT**
Dysgerminoma Yolk sac tumour Embryonal carcinoma Non-gestational choriocarcinoma Mixed GCT (at least two malignant histologies)
**Mature teratoma**
**Immature teratoma**
**Monodermal and somatic-type tumours arising from dermoid cysts** Struma ovarii (benign and malignant) Ovarian carcinoid Neuroectodermal type tumours Monodermal cystic teratomas Somatic neoplasms arising from teratoma

**Table 2:** Histological patterns observed in YST [6,10,24].

Histological Appearance of YST	Brief Description
Microcystic/reticular	Loose meshwork of cysts and tumour cells that have a ‘signet-ring’ morphology
Endodermal sinus (festoon)	Anastomosing network of labyrinthine-like spaces lined with tumour cells and Schiller-Duval bodies (glomeruloid structures)
Solid	Sheets of polygonal tumour cells
Alveolar-glandular	Large cystic/irregular alveolar spaces which are lined by single or multiple layers of columnar cells
Parietal	Tumour cells surround bands of PAS positive hyaline globules
Papillary	Pleomorphic tumour cells line papillae containing connective tissue fibrovascular cores
Polyvesicular vitelline	Cysts or vesicles lined by flat/columnar/cuboidal tumour cells
Hepatoid	Aggregates, cords or clusters of polygonal cells which resemble hepatocytes
Myxomatous	Myxoid stroma containing tumour cells with tubular/cords/glandular-like structures

Abbreviation: PAS, periodic acid positive.

**Table 3:** miRNAs and their targets in NEOC.

miRNA	Target
miR-199a-5p	Beclin 1 (BECN1) Podocalyxin-like (PODXL) MAF BZIP transcription factor B (MAFB)
miR-199a	Nuclear factor κB kinase subunit beta (IKKβ)
miR-202-3p	Forkhead Box L2 (FOXL2)
miR-506~514	Forkhead Box O1 (FOXO1)

**Table 4:** Currently active clinical trials of NEOC.

Trial Reference	Type of Trial	Target NEOC Patient Group Included	Interventions	Primary Endpoint	Recruitment Status
NCT04876456	Phase II	Refractory GCT	Cabozantinib	Clinical response according to RECIST	Recruiting
NCT04804007	Phase II	Relapsed GCT treated with HDCT + AuSCT	Etoposide vs. observation	12-month PFS	Recruiting
NCT04602377	Phase II	Advanced SCCOHT	Pembrolizumab + PAVEP for 6 weeks, followed by pembrolizumab alone vs. Pembrolizumab alone	Clinical response according to RECIST	Recruiting
NCT04602377	Phase II	GrCT	Onapristone ER vs. Onapristone ER + Anastrozole	Clinical response according to RECIST	Recruiting
NCT03067181	Phase III	Childhood and adult GCT	Active surveillance vs. Randomised trial of carboplatin vs. Cisplatin	OS and event-free survival 2 years post-enrolment	Recruiting
NCT02834013	Phase II	GCT	Nivolumab alone for PD-L1 amplified cohort vs. Nivolumab + ipilimumab for all other cohorts	Clinical response according to RECIST	Recruiting
NCT02429700	Phase III	SCST	Paclitaxel vs. BEP	5-year PFS	Recruiting
NCT01042522	Randomised phase II	GrCT, SLCT and SCST	Paclitaxel and carboplatin vs. BEP and cisplatin	10-year PFS	Active, not recruiting
NCT00788125	Phase I/II	GCT	Dasatinib with ifosfamide + carboplatin + etoposide	Maximum tolerated dose and toxicity of dasatinib	Active, not recruiting
NCT00788125	Phase I	HER overexpressed adult GCT	pNGVL3-hICD vaccine + sargramostim	Immune response and safety of vaccine	Active, not recruiting
NCT00432094	Phase II	Childhood + adult relapsed GCT	2 AuSCT with non-cross-resistant conditioning regimens vs. 1 AuSCT only	1-year OS	Active, not recruiting
NCT02582697	Phase III	Metastatic GCT	Standard BEP vs. Accelerated BEP	5-year PFS	Recruiting

Abbreviations: GCT, germ cell tumour; RECIST, Response Evaluation Criteria in Solid Tumours; HDCT, high dose chemotherapy; AuSCT, autologous stem cell transplant; PFS, progression-free progression; SCCOHT, Small Cell Ovarian Carcinoma of Hypercalcaemic Type; PAVEP, cisplatin, doxorubicin, vepeside, and cyclophosphamide; GrCT, Granulosa cell tumour; ER, extended release; OS, overall survival; PD-L1, Programmed death-ligand 1; SCST, sex cord stromal tumour; BEP, bleomycin, etoposide and cisplatin; SLCT, Sertoli-Leydig cell tumours.
